# Spatial distribution of COVID-19 patients in Sri Lanka

**DOI:** 10.1186/s12889-023-16481-2

**Published:** 2023-09-09

**Authors:** Lahiru Sandaruwan Galgamuwa, Nishan Madhushanka Liyanawahunge, Chamilka Gayashini Ratnayake, Navodi Mekala Hakmanage, Fahim Aslam, Samath D. Dharmaratne

**Affiliations:** 1 Department of Parasitology, Faculty of Medicine, Sabaragamuwa University of Sri Lanka, Ratnapura, Sri Lanka; 2 Department of Biochemistry, Faculty of Medicine, Sabaragamuwa University of Sri Lanka, Ratnapura, Sri Lanka; 3 Department of Chemistry, Faculty of Science, University of Colombo, Colombo 03, 00300 Sri Lanka; 4 Department of Statistics & Computer Science, University of Kelaniya, Kelaniya, 11600 Sri Lanka; 5 University of Bedfordshire, Luton, UK; 6 Department of Community Medicine, Faculty of Medicine, University of Peradeniya, Peradeniya, 20400 Sri Lanka; 7 Department of Global Health, School of Public Health, Institute for Health Metrics and Evaluation, University of Washington, Box 357230, Seattle, WA 98195 USA

**Keywords:** COVID-19, Spatial distribution, Seasonal variation, Sri Lanka

## Abstract

**Background:**

A new type of viral pneumonia, which has been named Coronavirus disease (COVID-19) began in Wuhan, China in late 2019 and has spread across the world since then. It has claimed more than 370 million confirmed cases and over 5.6 million deaths have been reported globally by the end of January 2022. This study aimed to analyze the trends, highly-nuanced patterns, and related key results relative to COVID-19 epidemiology in Sri Lanka.

**Methods:**

Data on COVID-19 from March 2020 to January 2022 were obtained from published databases maintained by the Epidemiology Unit of the Ministry of Health in Sri Lanka and information regarding populations in administrative districts was obtained from the Department of Census and Statistics, Sri Lanka. Descriptive spatiotemporal analysis and autocorrelations were analyzed using SPSS statistical software.

**Results:**

In Sri Lanka, the first case of COVID-19 was a Chinese national and the first local case was identified in the second week of March. As of 31^st^ of January 2022, a total of 610,103 COVID-19 cases had been recorded in the country, and 15,420 patients had died. At the beginning, the disease was mainly concentrated in the Western province and with time, it spread to other provinces. However, very low numbers of patients were identified in the North, Eastern, North Central, and Uva provinces until April 2021. The peak of COVID-19 occurred in August and September 2021 in all provinces in Sri Lanka. Then a decreasing trend of COVID-19 cases showed after September 2021.

**Conclusions:**

COVID-19 is an emerging public health problem in Western and Southern Sri Lanka where the population density is high. A decreasing trend of COVID-19 cases showed in all provinces after September 2021. Public awareness programs for the prevention and control of the disease in endemic regions are essential to reduce the incidence of this infection.

## Background

Pneumonia cases with no known cause were reported from Huanan Seafood Wholesale Market in China to be on the rise in December 2019 [1]. The cause of the pneumonia cases was identified as a novel type of coronavirus, a family of single-stranded, encapsulated RNA viruses that infect a wide range of animal species, including humans, and was dubbed coronavirus disease 2019 [2]. World Health Organization proclaimed a pandemic as a result of the severe global spread of SARS-CoV-2 and the resulting exceptionally high fatality rates. The COVID-19 epidemic has so far caused a considerable deal of harm to the world, particularly in terms of increasing poverty, mental health issues, and financial consequences [3]. Since the count is constantly changing and updating, it is challenging to estimate the precise number of cases and deaths that have been reported up to this point.

Basically, contact with or inhalation of infected droplets contributes to the spread of COVID-19. Following exposure, the typical symptoms include fever, sore throat, lethargy, cough, body pains, shortness of breath, malaise, and headache, which often appear 2 to 14 days later [4]. However, the majority of those who are sick are either asymptomatic or only have moderate symptoms, but they can spread the illness. Because the lung is the primary target of this virus, the majority of patients with the severe disease develop interstitial pneumonia, which can occasionally result in acute respiratory distress syndrome (ARDS), refractory respiratory failure, and death [5]. Although bacterial superinfection is a potential, the inflammatory response to viral infection during the acute phase of COVID-19 pneumonia causes lung damage [6]. Elderly and immunocompromised individuals with concomitant conditions such as diabetes, cardiovascular disease, chronic lung illness, and cancer are more likely to acquire the aforementioned problems [7].

Dynamics, patterns, and trends of disease spread through time and place can be identified by time series modeling for the spatial distribution of infectious illnesses. In order to explore the spatial distribution of infectious diseases, time series modeling techniques have been used in many studies. They have emphasized the significance of early disease intervention tactics [8, 9]. By combining the most significant prognostic indicators and determining their possible periodicity, time series modeling has the potential to improve the accuracy for estimating the number of COVID-19 cases [10, 11].

The first COVID-19 case in Sri Lanka was a Chinese national reported in January 2020. The first local patient was discovered in the second week of March [12]. Sri Lanka is currently seeing a third wave of illness recurrence, emphasizing the stubborn character of the outbreak, despite curfews being periodically implemented to preserve social distance while adhering to other public health measures strictly and regularly. Collaboration between public health organizations and medical organizations is essential to guaranteeing that the local government responds quickly to the disease’s mitigation. Furthermore, complete records and analysis of the data related to cases and fatalities linked to COVID-19 have the potential to serve as a model for national disease control policies. This study summarizes the trends, highly-nuanced patterns, and related key results relative to COVID-19 epidemiology in Sri Lanka.

## Methods

### Geography, population and climate

Sri Lanka is situated in the Indian Ocean between 5°55′ and 9°51′ N and 79°41′ and 81°53′ E, respectively. A total of 21.4 million people living in Sri Lanka in 65,610 km^2^ and the population density in Sri Lanka is 342 people per square kilometer in 2021 [13]. Sri Lanka is divided into nine provinces and twenty-five districts for administrative purposes. The majority of the island is made up of lowlands, with the center region of the island being hilly with an altitude range of 500 to 2500 m. In Sri Lanka, the typical annual temperature at low altitudes ranges from 26.5–28.5 °C, and as altitude rises, the temperature drops rapidly. The mean temperature at 1800 m above sea level is 15.9 °C.

**Fig. 1:**
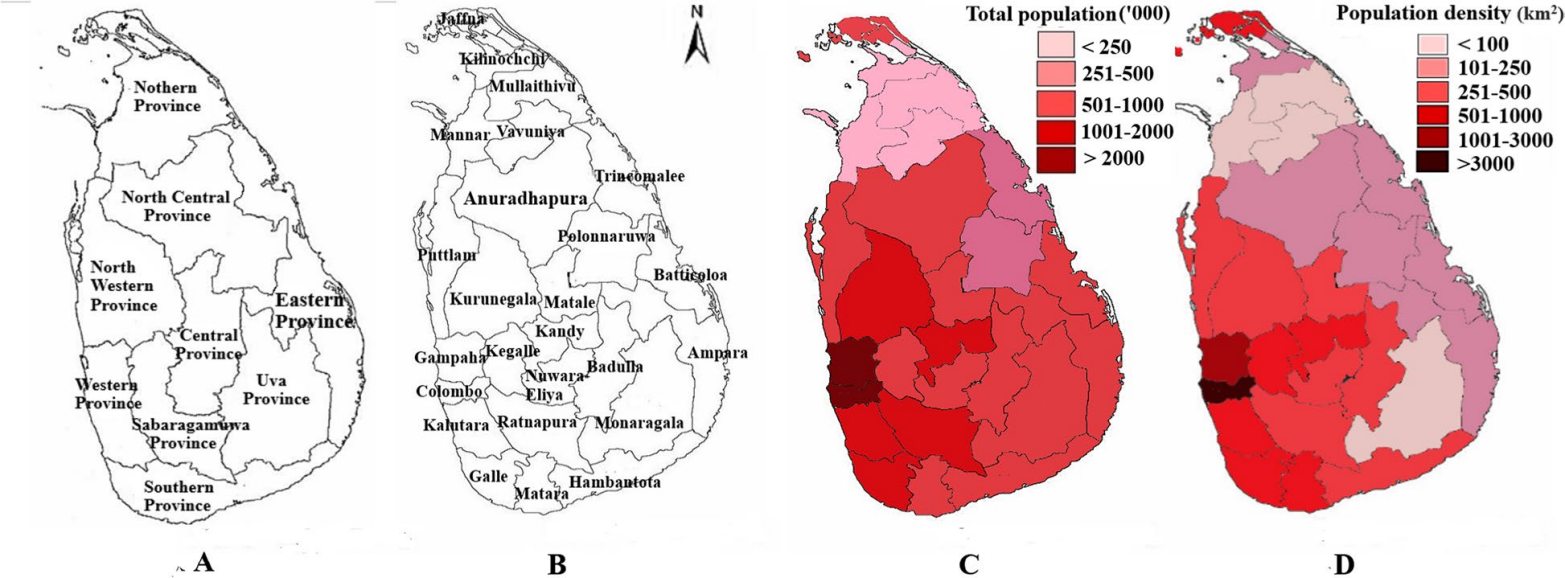
General information of Sri Lanka (**A** Provinces of Sri Lanka, **B** Administrative districts, **C** Total population by districts (in 2020), **D** Population density by districts in 2020))

Colombo metropolitan region is Sri Lanka’s most important administrative, commercial, and industrial region. Subsequently, Colombo is overwhelmingly the most densely populated district in Sri Lanka. The urban population in Sri Lanka in 2021 was 18.86 percent and the highest percentage was reported in districts in Western province [14]. The inhabitants of the coastal zone make up roughly 30 percent of the population of the island. In addition, 70 percent of tourist hotels and 62 percent of industrial units located in the coastal zone [15] (Fig. 1).

### Sampling of data

#### Data collection

The procedure was approved by the Ethics Review Committee, University of Peradeniya, Sri Lanka. COVID-19 raw data was obtained from the Epidemiological Department of Sri Lanka and the Health Statistics Unit of the Ministry of Health, Sri Lanka. COVID-19 confirmed data, Morbidity related data were obtained from reports and weekly reports published by the Epidemiology Unit and Health Statistics Unit of the Ministry of Health. Obtained raw data containing daily COVID-19 confirmed cases district-wise from 15^th^ November 2020 to 31^st^ January 2022. Furthermore, demographic data were collected from the Department of Census and Statistics of Sri Lanka for the study period [16].

### Statistical analysis

The number of confirmed cases of COVID-19 infection are collected for provinces in Sri Lanka from the official website from 11^st^ of March 2020 to 31^st^ January 2022. These daily based data were used to build predictive models for analysis. Time series information including the number of Covid-19 infected patients, trend and seasonal variations, and seasonal adjusted COVID-19 infected patients were analyzed in each province during the above-mentioned period known to be the COVID-19 third wave.

For statistical analysis, data were entered into a Microsoft Excel data sheet and analysed by SPSS software. To comprehend the fundamental causes of an observed time series, time series analysis entails creating models that characterize the time series. The COVID-19 infections were modeled and predicted using the Auto-Regressive Integrated Moving Average (ARIMA) model. Autocorrelation functions were used to find autoregressive functions. In time series analysis, the partial autocorrelation function (PACF) for seasonal data was used to determine the size of the lag in an autoregressive model. Every technique was used in compliance with the rules and regulations that were applicable.

### Trend analysis

A forecasting technique called a linear trend or trend-line model develops a linear relationship between time and the response variable. It is appropriate for time series when the local mean is rising steadily over the course of time. The simple linear regression model, in which the time index or another equally spaced series of numbers serves as the independent variable, has a specific case known as a linear trend. The Equation below represents the forecasting equation for the linear trend model.

$$Y\left(t\right)=\alpha +\beta T$$

where $\alpha$ and $\beta$ the regression coefficients whose values are calculated such that the sum of squared deviations from the data is the minimum.

#### Auto regressive integrated moving average

Autoregressive integrated moving average (ARIMA) is a combination of auto regressive (AR) and moving average (MA) models. AR is a regression model where the forecast value of a variable is a function of the past values of the variable as shown in Equation below.

$${Y}_{t}=\alpha +{\beta }_{1}{y}_{t-1}+{\beta }_{2}{y}_{t-2}+{\beta }_{3}{y}_{t-3}+\dots +{\beta }_{p}{y}_{t-p}+{\epsilon }_{t}$$

where $\alpha$ and $\beta$ the regression coefficients and the $\epsilon$ is the noise.

Instead of using the past values for the forecast as in AR, MA uses past forecast errors in a regression model as stated in Equation below.

$${Y}_{t}=\alpha +{\epsilon }_{\gamma }+{\varphi }_{1}{\epsilon }_{\gamma -1}+{\varphi }_{2}{\epsilon }_{\gamma -2}+{\varphi }_{3}{\epsilon }_{\gamma -3}+\dots +{\varphi }_{q}{\epsilon }_{\gamma -q}$$

where $\alpha$ and $\epsilon$ are the regression coefficients.

The AR and MA models combined together with differencing yields ARIMA as shown in Equation below.

$${Y}_{t}=\alpha +{\beta }_{1}{y}_{t-1}+{\beta }_{2}{y}_{t-2}+\dots +{\beta }_{p}{y}_{t-p}+{\epsilon }_{\gamma }+{\varphi }_{1}{\epsilon }_{\gamma -1}+{\varphi }_{2}{\epsilon }_{\gamma -2}+\dots +{\varphi }_{q}{\epsilon }_{\gamma -q}+{\epsilon }_{t}$$

## Results

### Spatial distribution of COVID-19

**Fig. 2:**
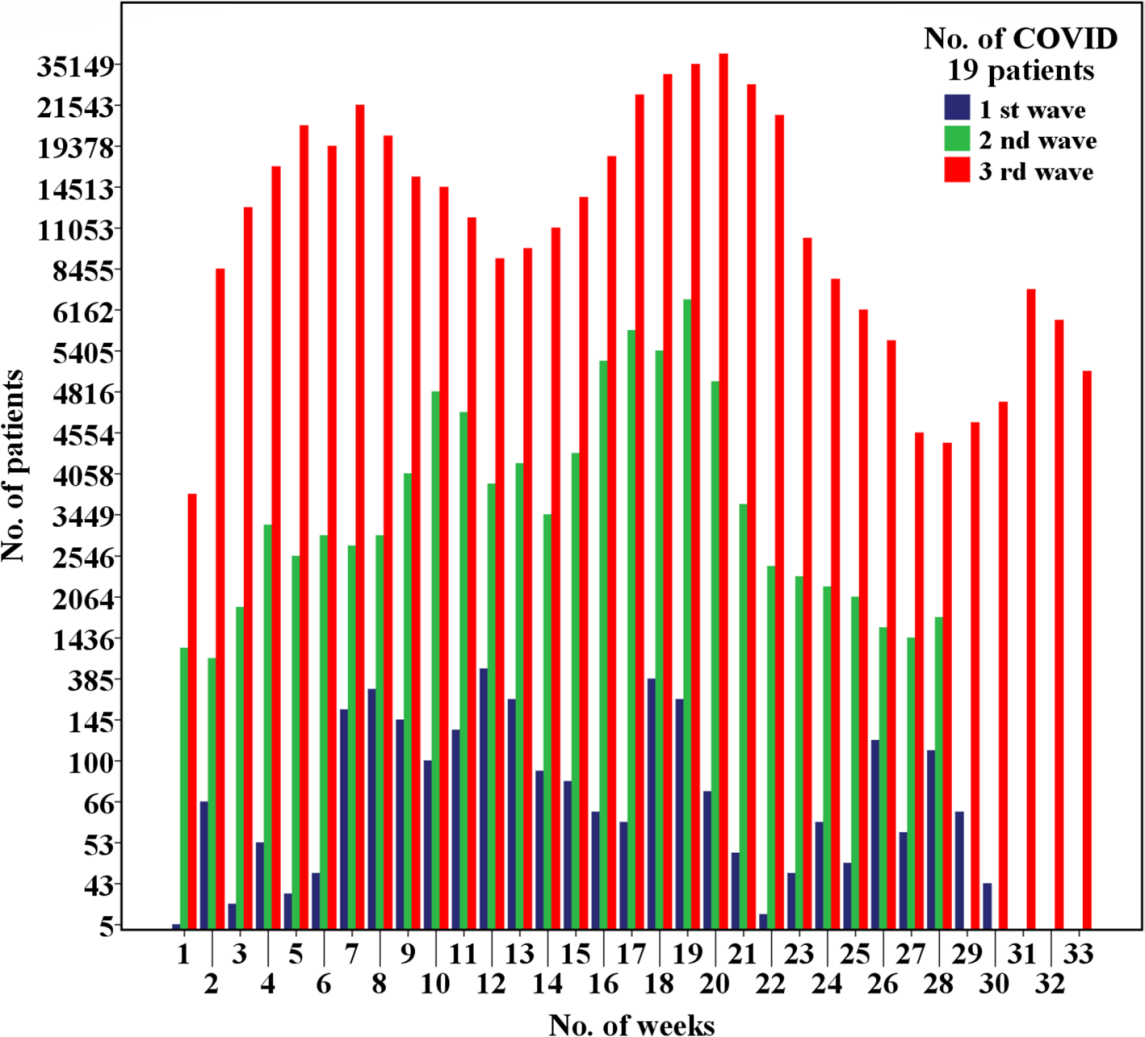
No. of COVID-19 patients according to 1^st^, 2^nd^, 3^rd^ waves and weeks

The first local COVID 19 patient was identified on 11^th^ March 2020. Since the total number of diagnosed COVID 19 patients reported to 31^th^ of January 2022 is 610,103. In Sri Lanka, 1^st^ COVID-19 wave was defined from 11^th^ March 2020 to 2^nd^ October 2020. Then second wave was considered from 3^rd^ October 202 to 14^th^ April 2021 and 3^rd^ wave started from 15^th^ April 2021 [11]. It was the deadliest wave and thousands of COVID-19 patients have been reported every day and the mortality rate was very high compare to the first and the second waves. This period was the highest incidence period as well as highest mortality rate from COVID 19 in Sri Lanka. Since October 2020, more than 10,000 cases have been newly identified monthly and the highest number recorded in August 2021. A total number of 3,396 COVID-19 patients and 13 deaths have been reported in the 1^st^ wave. During the second wave, the number of new cases increased to 92,341 cases, and there were nearly 591 recorded deaths. In the third wave the number of new cases increased to 505,427 and 14,816 deaths were documented by the end of January 2022 (Fig. 2).

Among the confirmed cases approximately 56.3% and 43.7% of individuals were male and female, respectively.

The first wave of COVID-19 characterized by 3396 total confirmed cases with 81.5% males and 18.5% females. In contrast to the other two waves, first wave has higher number of males confirmed cases. The confirmed cases during the second wave comprised slightly more females (60.0%) than males (40.0%), and most patients were aged 31 – 40 and 21–30 years. In contrast to the first wave, most cases occurred among migrant workers, followed by Sinhalese nationals and other foreigners. The third wave comprised slightly more males than females, and most patients were aged 21–30 and 31–40 years. Most patients were Sinhalese nationals, followed by migrant workers and other foreigners.

**Fig. 3:**
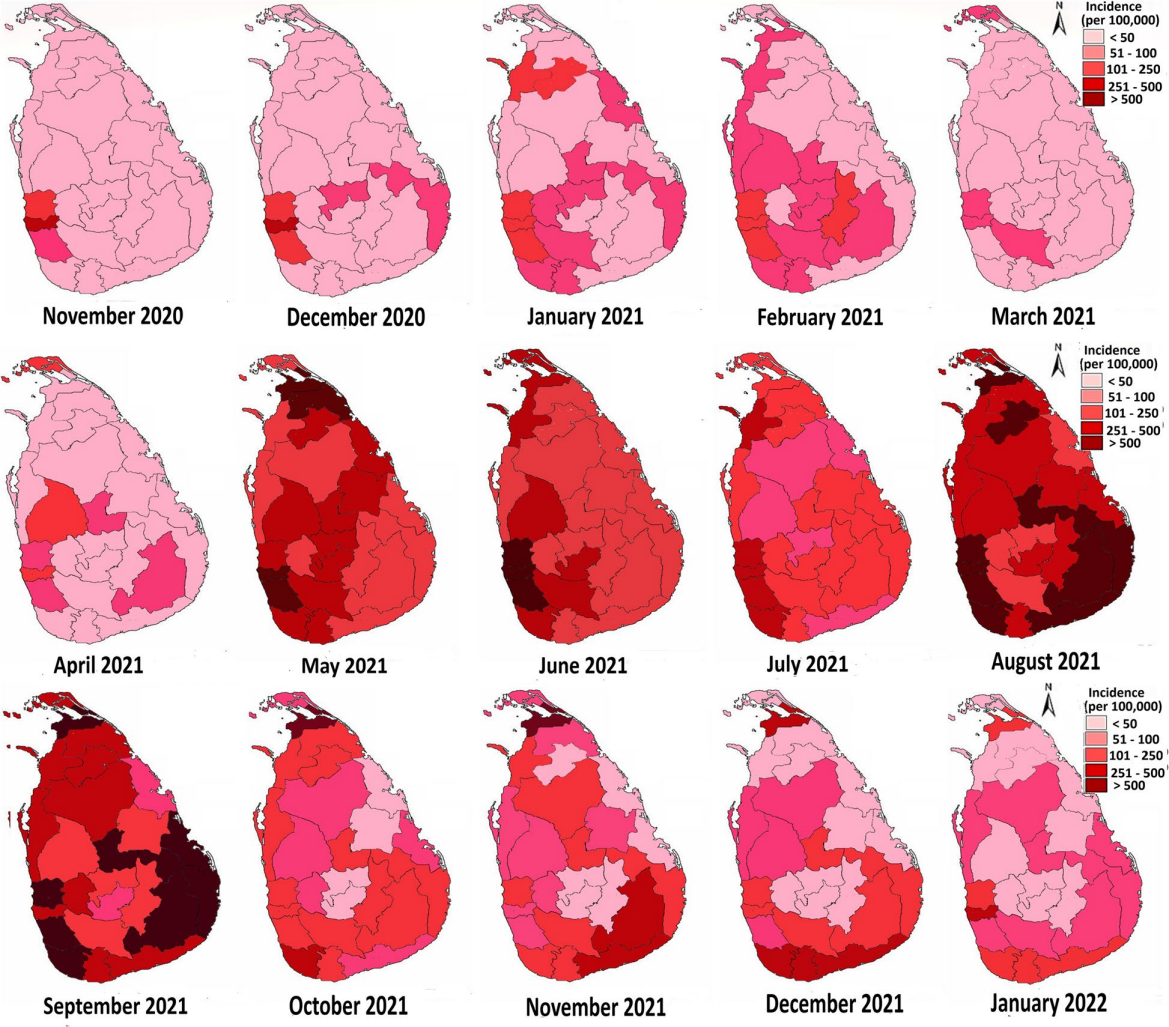
Incidence rates of COVID 19 in Sri Lanka per 100,000 people (From November 2020 to January 2022)

At the beginning of the study period, the disease was mainly concentrated in the Western province (Colombo, Gampaha and Kaluthara districts) where the population density is very high and with time, it spread to other districts (Fig. 3). However, very low numbers of patients were identified in the North, Eastern, North Central and Uva provinces until April 2021.

Although COVID-19 has been reported in all districts, Colombo, Gampaha, and Kalutara were the districts identified as high-risk areas. During the period of study, the highest number of COVID-19 patients was reported in the Colombo district (124,204) and it was 20.35% of all identified patients in this period. The second highest number of patients were identified in the Gampaha district (105,336; 17.27%) followed by Kaluthara district (54,992; 9.01%), Galle district (40,073; 6.57%) and Kurunegala district (29,271; 4.80%). When considering the third wave, a 3360-fold increase in COVID 19 patients was shown in Kilinochchi district followed by a 2346 fold increase in the Monaragala, and a 2245 fold increase in Hambanthota District compared to the second wave. In addition, the number of COVID-19 patients increased 1410 folds in Anuradhapura district and 1187 fold in the Batticoloa district in the third wave than the second wave [10].

Furthermore, the incidence of COVID-19 patients per 100,000 people in Colombo, Gampaha and Kaluthara districts rapidly increased from the beginning of the disease identification in Sri Lanka. In addition, a rapid increase of patients was observed in Kurunegala, Galle, Matara, Ratnapura, Jaffna and Kilinochchi from May and June 2021. Then number of patients were slightly decrease in July 2021. further in August and September 2021, large number of patients were reported from all over the country. During the period of August and September 2021, more than 500 patients per 100,000 people were identified in were reported in Batticoloa, Ampara, Matale, Monaragala, Kilinochchi, Hambanthota, Gampaha, Colombo and Kaluthara. In addition, the rate of COVID 19 incidence in Puttalam, Anuradhapura, Matara, Vavuniya, Jaffna, Mannar Mullaithivu districts has become more than 250 patients per 100,000 people during both months (Fig. 3).

### Seasonality and distribution of COVID 19 patients in Sri Lanka

**Fig. 4:**
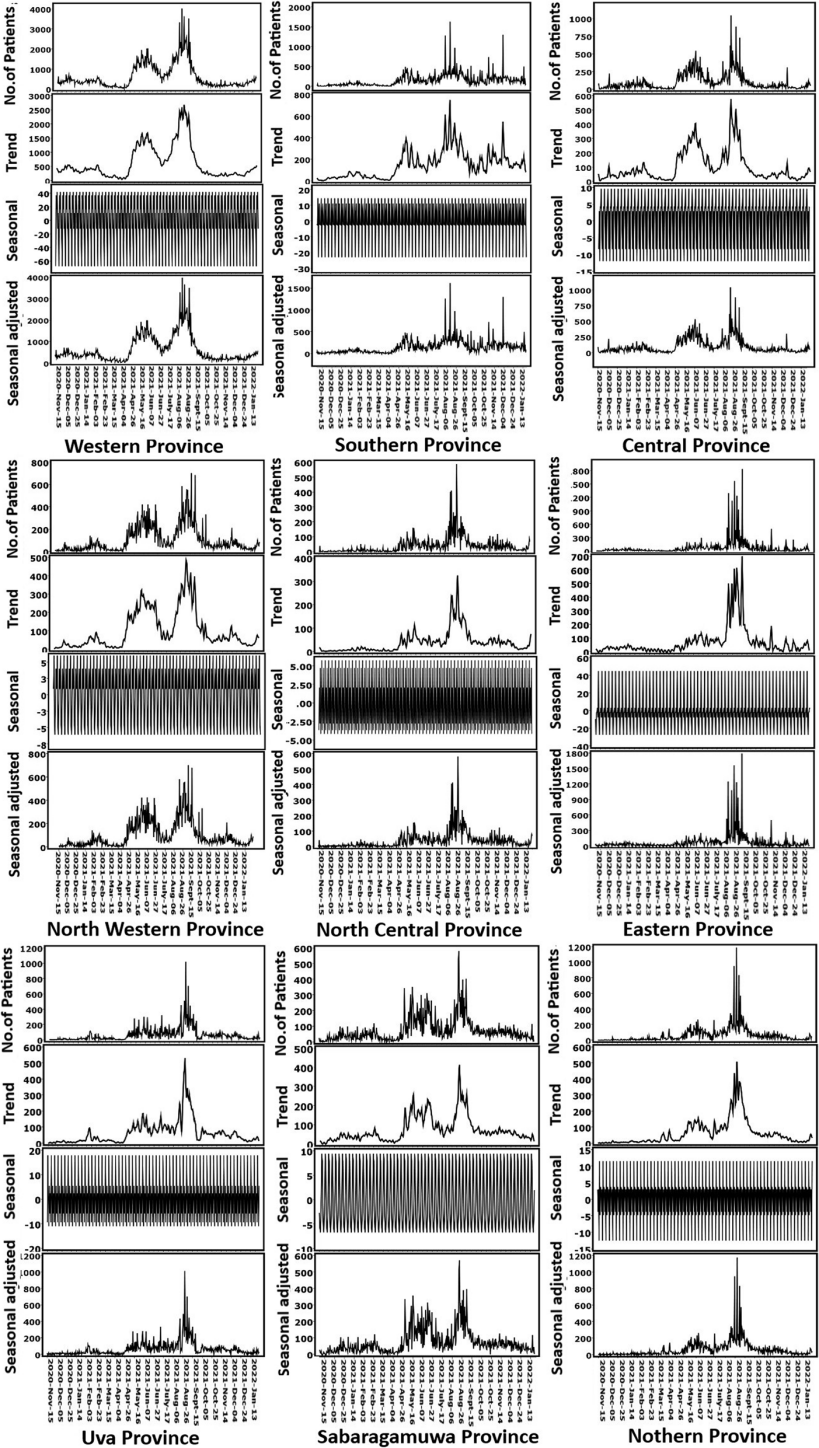
Trend analysis by provinces (From November 2020 to January 2022)

The top panel of Fig. 4 shows the distribution of the monthly cases of the disease cases of COVID- -19 in Sri Lanka between November 2020 to January 2022. The second panel is the trend component, which indicates two steady increases in COVID -19 cases in Western, Central, North Western and Sabaragamuwa provinces during the period of May and June 2021 and August and September 2021. Meanwhile, a sharp increasement of COVID-19 cases in Western and North Western provinces showed in April and August 2021. However, a decreasing trend of COVID-19 cases showed in all provinces after September 2021.

### Modeling and prediction of COVID-19 incidence

**Fig. 5:**
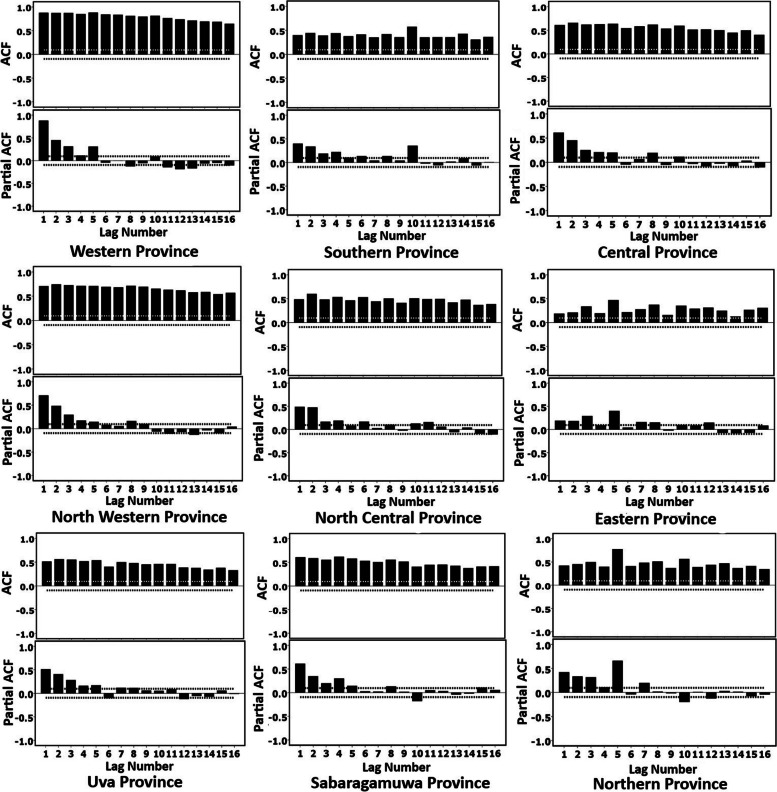
Autocorrelation plots by provinces

By assessing the autocorrelation and partial autocorrelation patterns, the nature of the time series can be understood. The autocorrelation plots in Western province, Central province, North Western province, Sabaragamuwa province and Uva province show small lags tend to be large and positive and slowly decrease as the lags increase. It suggests that the incidence of COVID-19 in those provinces has trends in a time series. In addition, Southern province, Eastern province, Northern province and North Central province have autocorrelations are larger for lags at multiples of the seasonal frequency than for other lags. The slow decrease in the ACF as the lags increase is due to the trend, while the “scalloped” shape is due the seasonality. Therefore, these plots confirmed that in time series of Southern province, Eastern province, Northern province and North Central province has both a trend and seasonality (Fig. 5).

Further, partial autocorrelation function (PACF) shows a significant autocorrelation at lag 1 and several lags in all provinces concluding no evidence of non-randomness process (Fig. 5). However, the PACF plots in Southern province, Eastern province, Northern province and North Central province have several significant lags followed by a drop in PACF values and they become insignificant.

## Discussion

Until COVID-19 became a global problem, public health awareness of viral diseases and its impact on health was underestimated for several decades. The findings suggest that approximately 600,000 verified COVID-19 cases have been reported during the period of January 2020 to January 2022, along with more than 16,000 fatalities in Sri Lanka. Since more instances have been found as a result of higher testing rates, variations between the identified and actual number of cases are mostly dependent on the depth of testing and diagnosis.

There have been three COVID-19 waves in Sri Lanka, the initial wave of which was driven by the Wuhan variety and resulted in 3,396 cases and 13 fatalities. The B.1.411 variant-driven second wave lasted from October 2020 through April 2021. Three peaks made up the third wave, with the Alpha version driving the first and the Delta and Omicron variants coming after. In the first wave (January 2020–October 2020), number of COVID-19 patients in Sri Lanka increased gradually but progressively. Public health social measures, including mask-wearing, movement restrictions, and public transport restrictions contributed to prevent large spreading in the first wave in Sri Lanka. Additionally, the government implemented a tight strategy of case discovery, contact identification, quarantine, travel limitations, and patient isolation [17, 18].

When COVID-19 first hit Sri Lanka, it only affected the Western province, which has the highest population density in the country. Localities with high concentrations of urban poor people living in cramped housing and areas appear to be more vulnerable than those with greater resources [19]. In Sri Lanka, large number of people are living with low socio-economic conditions and overcrowding in urban areas. COVID-19 spread fast among these communities because of difficult contact tracing and poor personal hygiene. This was proven that large number of COVID-19 patients have been identified from low socio communities in Colombo and suburb metropolitan areas in the first and second waves. Epidemiological simulations indicated that the disease would spread more quickly in urbanized regions than in rural ones in the absence of mitigation measures [20].

The second wave (October 2020–April 2021) has been linked to employees working in the private sector and sellers in one of the largest fish markets in Sri Lanka close to Colombo [21]. The majority of confirmed cases were migratory workers moving between districts [22]. Cluster communities and direct contact with a previously confirmed patient were the biggest risk factors. The close proximity of the immigrant population to the local community and the lack of personal precautions to stop illness spread were two factors that contributed to the outbreak. Since then, the Sri Lankan government has put regulations in place to help migrant workers who reside there. To effectively contain and control the COVID-19 pandemic, early and prompt interventions as well as reinforced social distance measures should be put into place [23].

Beginning in 2021, B.1.1.7 (alpha) was discovered, and since then, Sri Lanka has seen a sharp rise in its prevalence. B.1.1.7 lineage had entirely replaced the circulating B.1.411 due to its higher transmissibility by the end of April 2021. The outbreak, caused by the alpha variant, existed from April 2021 to June 2021, with daily reported cases reaching 9,950 [24]. Both the number of illnesses and the number of fatalities increased exponentially as a result of the introduction and dissemination of the B.1.1.7.

The delta variant and its sublineages were responsible for the largest SARS-CoV-2 outbreak in Sri Lanka between July and October 2021 [25]. The PCR positivity rates increased above 30% with case fatality rates reaching 6.35% [25]. Two other delta sub lineages, AY.28 and AY.104, were the most prevalent variants found during this time period in addition to the delta parental lineage B.1.617.2 [26]. Several SARS-CoV-2 virus BA.1, BA.2, and B.1.1.529 sublineages were found in Sri Lanka during the final week of December 2021 and the first week of January 2022. The most current form, B.1.1.529, later known as Omicron, was discovered in November 2021 in South Africa. After February 2022, Omicron was be the dominant variety due to its rapid expansion and consequently higher hospitalization rates.

All patients are initially transported by ambulance to the closest designated hospital for care and confirmatory testing. Patients who are experiencing severe acute respiratory distress must receive treatment right once and be transferred to specialized isolation or intensive care facilities. All of the COVID-19 patients’ close associates were also examined. Those who do not exhibit symptoms are subject to a 14-day quarantine. Later, all asymptomatic patients from intermediate care facilities and secondary care facilities who did not require hospitalization were instructed to undergo home-based integrated care in an effort to make hospital facilities more accessible to those who needed them the most.

The vaccination campaign in Sri Lanka began January, 2021, with the immunization of frontline healthcare workers as well as key personnel engaged in pandemic prevention efforts and the delivery of critical services. After that, the immunization was made available to the broader public using a risk-based strategy, prioritizing the elderly and people with co-morbid conditions.

Traditional media, including as television (TV) channels, radio stations, newspapers, as well as social media platforms, have a considerable impact on the community’s ability to get accurate information during this time of crisis [27]. The mass media does a good job of promoting healthy habits through various commercials to raise public awareness and stop the development of the disease in the nation. For instance, visual or video messages are the ideal source of knowledge for populations with low literacy rates and adults who are less familiar with reading.

After health restrictions were loosened over the Sinhala and Tamil New Year in April 2021, there was an abrupt rise in COVID-19 infections. It eventually spread from the Western region in Sri Lanka to other districts, and the number of cases has significantly grown. The health system was overworked and the mortality rate spiked in August and September 2021 as a result of the enormous number of critically unwell people who were reported in a short period of time. Since August 2021, a remarkably high fatality rate has been observed in the nation due to the highly contagious Delta variety [28]. By August 2021, Sri Lanka became the fourth-highest population in the world in terms of daily fatalities.

The climax of COVID-19 happened in August and September of 2021. This rise was mostly caused by the virus’s Delta form emerging in Sri Lanka and many other nations. The western province of Sri Lanka reported the presence of a delta variation with four mutations by the middle of 2021, and the prevalence of the mutant delta appears to rise over time, suggesting that it may be more contagious than the original delta [29].

The delta form, which became prevalent at the end of August 2021, has replaced the earlier dominant alpha variant. The Government again imposed an inter-provincial travel ban in August 2021 in response to repeated recommendations from health experts to lock down the country as the more lethal Covid-19 Delta variant spread like wildfire throughout Colombo and the rest of the country, resulting in a 48 percent increase in the number of fatalities [30]. Due to COVID-19’s high transmission potential, a delayed response may have sped up the disease’s spread [31].

Large number of patients have been reported in Western province in all three waves because the population density is higher than in other districts and high number of industrial zones located in the Western province. Therefore, the interconnection between people is much higher than in other districts. It is a crucial factor to spread COVID-19 virus among large number of people in Western province. Due to frequent migration from Western province to other adjacent provinces (Sabaragamuwa, Southern and North Western), the number of patients increases gradually in Sabaragamuwa, Southern and North Western provinces in the second and third waves.

Early in 2020, Sri Lanka’s government formally launched a zero-COVID approach, blocking the novel coronavirus from entering the country through strong border security and taking active action to stop any local outbreaks by enforcing stringent contact tracking and isolation. However, errors in adherence to isolation protocols resulted in broad community transmission that went unnoticed for months in the absence of significant levels of PCR testing for symptomatic individuals in neighborhood healthcare institutions [32]. This transmission outpaced the nation’s capacity for testing, tracing, and isolation by the end of 2020. The testing rates required to maintain the COVID-19 eradication plan soon became apparent as being difficult to achieve [33].

Authorities tacitly gave up on their plan to end the pandemic in the face of this difficulty. They reduced attempts to find new cases and contacts and relaxed border procedures. The Sri Lankan government used a variety of social isolation tactics from the start of the COVID-19 epidemic until the beginning of 2022 to lessen the spread of the new coronavirus. These measures included closing schools and universities as well as placing restrictions on events like weddings. Depending on the number of cases around the nation, these restrictions were either loosened or strengthened during 2021 [32]. Attempts were also made to persuade office workers in the public and private sectors to work from home, especially during the delta wave, but the majority of Sri Lankan workers are not office workers and were unable to do so [34].

Many workers in the private sector had low access to employment protection or other social protection benefits related to their jobs, which makes them more susceptible to COVID-19 [35]. Because it is more difficult to preserve social distance in metropolitan areas than it is in rural ones, the number of COVID-19 applicants has increased quickly [36].

The highest number of patients have been reported in the third wave which may be caused by a lack of social distancing measures and public health initiatives and increasing diagnostic investigations [37]. The government-imposed lockdown, which was in effect from August 2021 to October 2021 guaranteed public discipline and was a major factor in lowering the death toll and the number of sick people [38]. The final effect of COVID-19 on Sri Lankans is determined by the public’s support for the immunization program and preventive measures.

The COVID-19 epidemic has created a conflict between promoting the economy and regulating public health [24]. High-risk groups should be vaccinated first in order to reduce the danger of transmission and provide universal protection against emerging variations. It is anticipated that as vaccination rates increase, viral circulation will decline and fewer mutations will occur. Sri Lankan government began immunizing the populace in mid-January 2021, giving older age groups and frontline medical workers priority. As of September 2021, more than 10 million people had received the first dose and half of them had received the second dose of the COVID-19 vaccination [39].

It has been discovered that social isolation policies have slowed COVID-19’s development rate and that, in the absence of treatments, the disease may spread exponentially [40]. Due to the restricted capacity for diagnostic testing, the true epidemic growth rate may differ from the reported epidemic growth rate [41]. A time series forecasting method can be used to predict future COVID-19 cases by identifying a lead-lag impact between the confirmed cases in various regions [10].

The COVID-19 vaccine is regarded as essential for preventing and controlling the corona infections because vaccination is one of the most effective and economical preventive measures to control the infectious diseases. The public is given the vaccines free of charge by the government. With the help of government employees, medical experts, and a robust supply chain that included cold storage facilities, a dependable routine vaccination network was established in Sri Lanka. The likelihood of symptomatic infections is greatly reduced after vaccination because it only produces partial immunity. In order to reduce the epidemic without further strict lockdowns and contact restrictions, vaccination coverage of 75%–80% is required.

Depending on the contact-reducing measures in place, the vaccination’s effects on incidence and mortality will vary. Vaccination programs, strict travelling bans and awareness of precaution measures mainly contributed to reduce the incidence of COVID 19 after October 2021. However loosen the restrictions for cultural events and air travel as well as rapid expansion of Omicron variant increase the number of patients again from the end of 2021.

This study is mainly focus for population analysis. Therefore, we could not investigate determinants at the individual level in the current study. Regardless, the main objective of this study is to estimate representative data at the country level that might be applied by national public health organizations.

## Conclusions

In Sri Lanka’s, COVID-19 incidence is high in Western and Southern regions, where there is a significant population density. Initiatives to educate the public and healthcare professionals about the disease will be crucial to halting its growth in Sri Lanka. When adopting public health initiatives, it is crucial to consider the unique sociocultural and medical demands of the communities and to balance any potential drawbacks with the benefits for the broader populace. Following the attainment of a sufficient immunization rate, additional study is required to evaluate the efficacy of public health initiatives.

## Data Availability

The datasets used and/or analysed during the current study are publicly available. COVID-19 Situation Report (epid.gov.lk).

## Abbreviation

COVID-19: Coronavirus disease-19
ACF: Autocorrelation function
PACF: Partial autocorrelation
WHO: World Health Organization
